# Crystal structure of *N*′-[bis­(ethyl­sulfan­yl)methyl­idene]-2-hy­droxy-4-meth­oxy­benzohydrazide

**DOI:** 10.1107/S2056989015021271

**Published:** 2015-11-21

**Authors:** Paras Nath, Manoj K. Bharty, Rahul Chaurasia, Sanyucta Kumari, Sushil K. Gupta

**Affiliations:** a Department of Chemistry, Banaras Hindu University, Varanasi 221 005, India; b School of Studies in Chemistry, Jiwaji University, Gwalior 474 011, India

**Keywords:** crystal structure, di­thio­carbazate, hydrogen bonding, ester

## Abstract

In the title compound, C_13_H_18_N_2_O_3_S_2_, the amide group is in the plane of the ­benzoyl ring with a C—N—N—C torsion angle of 177.63 (12)°. The two di­thio­ate groups are in an *anti* conformation [torsion angles = 173.68 (8) and −9.98 (10)°]. An intra­molecular N—H⋯O hydrogen bond is observed. In the crystal, an O—H⋯O hydrogen bond and a weak C—H⋯O contact involving the same acceptor atom generate an *S*(6) ring motif and give rise to chains along [010].

## Related literature   

For *S*-alk­yl/aryl esters of di­thio­carbaza­tes that form metal complexes, see: Ali *et al.* (2008 ▸); Singh *et al.* (2010 ▸, 2012 ▸). For their biological properties, see: Bharti *et al.* (2000 ▸). For cyclization of potassium salts of *N*-(aro­yl)hydrazine carbodi­thio­ates, see: Singh *et al.* (2008 ▸, 2009 ▸); Bharty *et al.* (2012 ▸). For bidentate, tridentate and multidentate esters, see: Wang *et al.* (2002 ▸); Tarafder *et al.* (2000 ▸); Ali *et al.* (2001 ▸). For related structures, see: Jasinski *et al.* (2010 ▸); Butcher *et al.* (2007 ▸); Tayamon *et al.* (2012 ▸). 
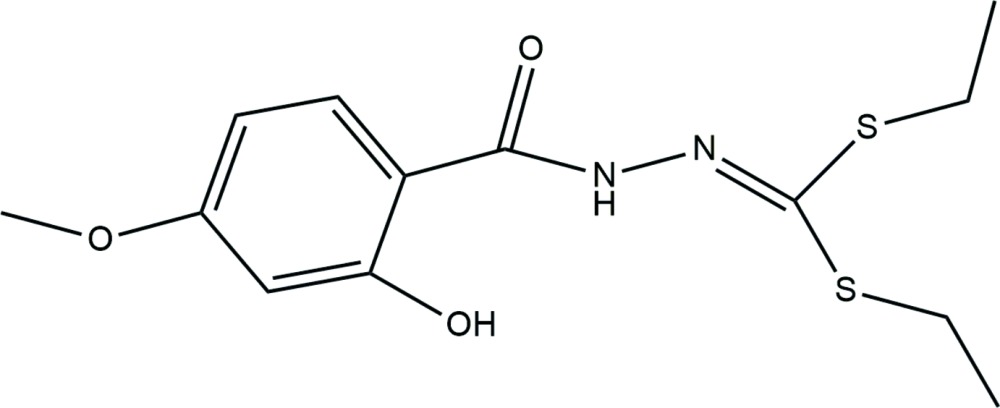


## Experimental   

### Crystal data  

C_13_H_18_N_2_O_3_S_2_
*M* *_r_* = 314.41
Monoclinic, 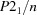

*a* = 8.479 (2) Å
*b* = 12.894 (3) Å
*c* = 13.843 (3) Å
β = 104.156 (10)°
*V* = 1467.5 (6) Å^3^
*Z* = 4
Mo *K*α radiation
μ = 0.37 mm^−1^
*T* = 293 K
0.2 × 0.2 × 0.2 mm

### Data collection  

Rigaku Mercury 375R diffractometer
Absorption correction: multi-scan (*CrystalClear-SM Expert*; Rigaku, 2011 ▸) *T*_min_ = 0.938, *T*_max_ = 1.000
12952 measured reflections
2685 independent reflections
2530 reflections with *I* > 2σ(*I*)
*R*_int_ = 0.027

### Refinement  

*R*[*F* ^2^ > 2σ(*F* ^2^)] = 0.029
*wR*(*F* ^2^) = 0.077
*S* = 1.08
2685 reflections
192 parameters
H atoms treated by a mixture of independent and constrained refinement
Δρ_max_ = 0.28 e Å^−3^
Δρ_min_ = −0.27 e Å^−3^

### 

Data collection: *CrystalClear-SM Expert* (Rigaku, 2011 ▸); cell refinement: *CrystalClear-SM Expert*; data reduction: *CrystalClear-SM Expert*; program(s) used to solve structure: *SHELXS97* (Sheldrick, 2008 ▸); program(s) used to refine structure: *SHELXL2014* (Sheldrick, 2015 ▸); molecular graphics: *SHELXTL* (Sheldrick, 2008 ▸); software used to prepare material for publication: *SHELXTL*.

## Supplementary Material

Crystal structure: contains datablock(s) I. DOI: 10.1107/S2056989015021271/jj2195sup1.cif

Structure factors: contains datablock(s) I. DOI: 10.1107/S2056989015021271/jj2195Isup2.hkl

Supporting information file. DOI: 10.1107/S2056989015021271/jj2195Isup3.cml

**Fig. 1.**
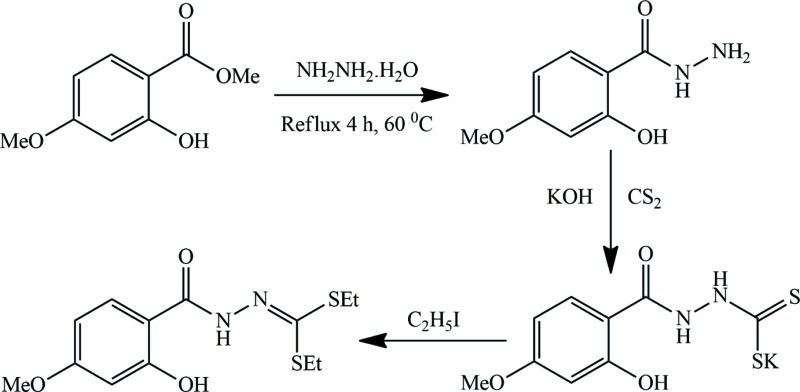
A reaction scheme showing the synthesis of the title compound, C13H18N2O3S2.

13 18 2 3 2 . DOI: 10.1107/S2056989015021271/jj2195fig1.tif
A reaction scheme showing the synthesis of the title compound, C_13_H_18_N_2_O_3_S_2_.

**Fig. 2.**
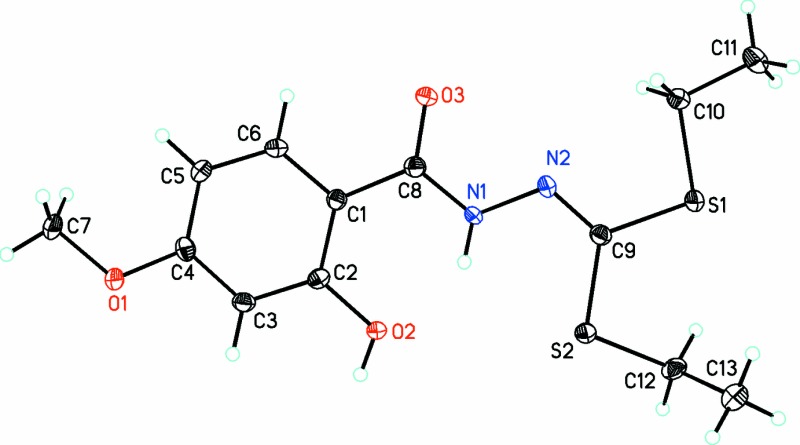
Molecular structure of the title compound, C13H18N2O3S2, showing 50% probability displacement ellipsoids.

13 18 2 3 2 . DOI: 10.1107/S2056989015021271/jj2195fig2.tif
Mol­ecular structure of the title compound, C_13_H_18_N_2_O_3_S_2_, showing 50% probability displacement ellipsoids.

**Fig. 3.**
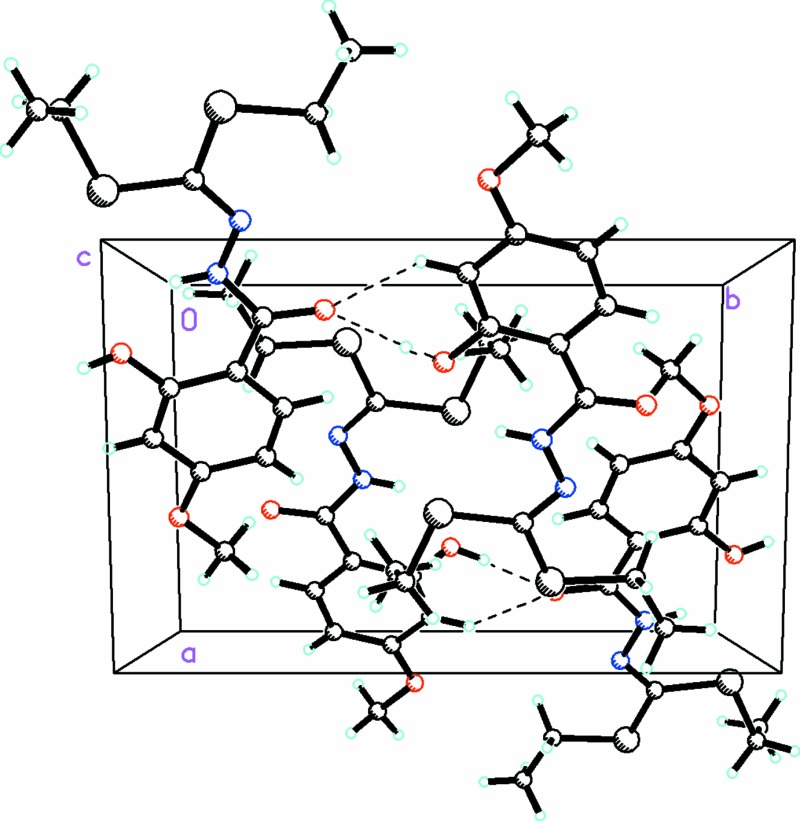
Molecular packing of C13H18N2O3S2 viewed along the c-axis. Dashed lines indicate intermolecular hydroxyl O—H···Ocarbonylhydrogen bonds and weak phenyl C—H···Ocarbonyl interactions.

13 18 2 3 2 c H carbon­yl carbon­yl . DOI: 10.1107/S2056989015021271/jj2195fig3.tif
Mol­ecular packing of C_13_H_18_N_2_O_3_S_2_ viewed along the *c*-axis. Dashed lines indicate inter­molecular hydroxyl O—*H*⋯O_carbon­yl_hydrogen bonds and weak phenyl C—H⋯O_carbon­yl_ inter­actions.

CCDC reference: 1431260

Additional supporting information:  crystallographic information; 3D view; checkCIF report

## Figures and Tables

**Table 1:** Hydrogen-bond geometry (Å, °)

*D*—H⋯*A*	*D*—H	H⋯*A*	*D*⋯*A*	*D*—H⋯*A*
O2—H2*A*⋯O3^i^	0.82 (2)	1.80 (2)	2.6118 (15)	177 (2)
N1—H1*A*⋯O2	0.836 (19)	1.957 (19)	2.6384 (16)	137.9 (17)
C3—H3*A*⋯O3^i^	0.93	2.52	3.1975 (18)	130

Symmetry code: (i) 
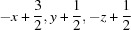
.

## supplementary crystallographic information

### S1. Comment

Nitro­gen-sulfur donor chelating agents such as di­thio­carbaza­tes and their S-alkyl/aryl esters have shown inter­esting biological properties (Bharti *et al.* (2000). They form large number of metal complexes with novel structural features (Ali *et al.*, 2008; Singh *et al.*, 2010; Singh *et al.*, 2012). The S-alkyl/aryl esters derived from potassium salts of N-aroylhydrazine carbodi­thio­ates have been found to be more stable towardscyclization (Singh *et al.*, 2008) and form 1,3,4-oxa­diazole-2-thio­nes in the presence of acid or base (Singh *et al.*, 2009; Bharty *et al.*, 2012). The above esters behave as bidentate, tridentate or multidentate chelating agents with hetero donor atoms (Wang *et al.*, 2002; Tarafder *et al.*, 2000; Ali *et al.*, 2001). In view of the importance of the title compound, (I), herein we report its synthesis and crystal structure.

In the title compound, C_13_H_18_N_2_O_3_S_2_, the sum of the angles around C9 (120.0°) and the S1/C9/S2 bond angle of 117.94 (8)° indicate a nearly planar *sp*^2^ hybridized carbon atom (Fig. 2). The amide group is in plane to the benzoyl ring with a C8—N1—N2—C9 torsion angle of 177.63 (12)°, in contrast to the values in related structures (Jasinski *et al.* (2010); Butcher *et al.* (2007); Tayamon *et al.* (2012)). The two di­ethyl di­thio­ate groups are in an *anti* conformation with respect to each other, as reflected by torsion angles C10/S1/C9/S2 of 173.68 (8)° and S1/C9/S2/C12 of – 9.98 (10)°. The N2 atom in the amide linkage possesses distorted tetra­hedral geometry (C9/N2/N1 = 116.59 (12)° while the N1 atom is almost planar (sum of bond angles 359.6°). The C8—N1 and C9—N2 bond lengths (1.3448 (18) Å and 1.2837 (18) Å) lie between typical C–N and C═N values owing to the extensive delocalization of π electron density over the C9/N2/N1/C8 linkage.

In the crystal, an inter­molecular O2–H2A···O3 hydrogen bond and weak inter­molecular C3–H3A···O3 contact in bifurcated bonding arrangement (Fig. 3) generate an *S*(6) ring motif (Table 1). Intra­molecular N1–H1A···O2 hydrogen bonds are also observed.

### S2. Experimental

Potassium 2-(2-hy­droxy-4-meth­oxy­benzoyl)­hydrazinecarbodi­thio­ate was prepared by adding carbon di­sulfide (20.0 mmol, 1.50 mL) to a solution of 4-meth­oxy­salicylic acid hydrazide (20.0 mmol, 3.65 g) and potassium hydroxide (20.0 mmol, 1.12 g) in ethanol (30 mL), then stirring the reaction mixture for 2 h (Fig. 1). The solid separated was filtered off, washed with ethanol and dried in *vacuo*. Ethyl iodide (20.0 mmol, 1.60 mL) was added drop-wise to a suspension of potassium 2-(2-hy­droxy-4-meth­oxy­benzoyl)­hydrazinecarbodi­thio­ate (10.0 mmol, 2.96 g) in ethanol (20 mL) and stirring the reaction mixture for a period of 3–4 h. The resulting yellow solution was concentrated and acidified with dilute CH_3_COOH (20% v/v) which yielded a yellow precipitate, washed with water and dried in *vacuo*. Yellow crystals of (I) (m.p. 427–429 K), suitable for X-ray analysis were obtained by slow evaporation of the methanol solution over a period of 9–10 days (yield 60%): Anal. Calc. for C_13_H_18_N_2_O_3_S_2_ (%): C, 49.61; H, 5.77; N, 8.91; S, 20.40. Found: C, 49.24; H, 5.85; N, 8.86; S, 20.12.

### S3. Refinement

The H atoms bonded to N1 and O2 were located in a difference Fourier map and refined freely; N1–H1A = 0.836 (19) Å and O2–H2A = 0.82 (2)Å. Other H atoms were positioned geometrically and allowed to ride on their parent atoms, with C—H = 0.93–0.97 Å, and with *U*_iso_(H) = 1.2–1.5*U*_eq_(C).

### Crystal data

**Table:**

C_13_H_18_N_2_O_3_S_2_	*F*(000) = 664
*M**_r_* = 314.41	*D*_x_ = 1.423 Mg m^−^^3^
Monoclinic, *P*2_1_/*n*	Mo *K*α radiation, λ = 0.71073 Å
*a* = 8.479 (2) Å	Cell parameters from 4529 reflections
*b* = 12.894 (3) Å	θ = 3.0–27.5°
*c* = 13.843 (3) Å	µ = 0.37 mm^−^^1^
β = 104.156 (10)°	*T* = 293 K
*V* = 1467.5 (6) Å^3^	Prism, colorless
*Z* = 4	0.2 × 0.2 × 0.2 mm

### Data collection

**Table:**

Rigaku Mercury 375R diffractometer	2685 independent reflections
Radiation source: Sealed Tube	2530 reflections with *I* > 2σ(*I*)
Detector resolution: 13.6612 pixels mm^-1^	*R*_int_ = 0.027
ω scans	θ_max_ = 25.3°, θ_min_ = 3.0°
Absorption correction: multi-scan (*CrystalClear-SM Expert*; Rigaku, 2011)	*h* = −10→10
*T*_min_ = 0.938, *T*_max_ = 1.000	*k* = −15→15
12952 measured reflections	*l* = −16→16

### Refinement

**Table:**

Refinement on *F*^2^	Primary atom site location: structure-invariant direct methods
Least-squares matrix: full	Secondary atom site location: difference Fourier map
*R*[*F*^2^ > 2σ(*F*^2^)] = 0.029	Hydrogen site location: mixed
*wR*(*F*^2^) = 0.077	H atoms treated by a mixture of independent and constrained refinement
*S* = 1.08	*w* = 1/[σ^2^(*F*_o_^2^) + (0.0391*P*)^2^ + 0.8434*P*] where *P* = (*F*_o_^2^ + 2*F*_c_^2^)/3
2685 reflections	(Δ/σ)_max_ < 0.001
192 parameters	Δρ_max_ = 0.28 e Å^−^^3^
0 restraints	Δρ_min_ = −0.27 e Å^−^^3^

### Special details

**Table:**

Experimental. IR (selected, KBr): 3320 [ν(O–H)], 3276 [ν(N–H)], 1634 [ν(C═ O)], 1094 [ν(N–N)], 876 [ν(C═S)] cm^-1. 1^H NMR (DMSO-d_6_); δ [p.p.m.] = 12.26 (s, 1H, OH), 9.77 (s, 1H, NH), 7.80–6.40 (m, 3H, C_6_H_3_, phenyl), 3.82 (s, 3H, –OCH_3_), 3.06 (q, 4H, CH_2_), 1.44 (t, 6H, CH_3_). ^13^C NMR (DMSO-d_6_) δ [p.p.m.] = 168.3 (C4), 163.3 (C8), 162.0 (C2), 157.9 (C9), 132.1 (C6), 128.2 (C5), 106.8 (C3), 55.2 (C7), 27.1 (C10, C12), 14.4 (C11, C13).
Geometry. All e.s.d.'s (except the e.s.d. in the dihedral angle between two l.s. planes) are estimated using the full covariance matrix. The cell e.s.d.'s are taken into account individually in the estimation of e.s.d.'s in distances, angles and torsion angles; correlations between e.s.d.'s in cell parameters are only used when they are defined by crystal symmetry. An approximate (isotropic) treatment of cell e.s.d.'s is used for estimating e.s.d.'s involving l.s. planes.
Refinement. Refinement on *F*^2^ for ALL reflections except those flagged by the user for potential systematic errors. Weighted *R*-factors *wR* and all goodnesses of fit *S* are based on *F*^2^, conventional *R*-factors *R* are based on *F*, with *F* set to zero for negative *F*^2^. The observed criterion of *F*^2^ > σ(*F*^2^) is used only for calculating -*R*-factor-obs *etc*. and is not relevant to the choice of reflections for refinement. *R*-factors based on *F*^2^ are statistically about twice as large as those based on *F*, and *R*-factors based on ALL data will be even larger.

### Fractional atomic coordinates and isotropic or equivalent isotropic displacement parameters (Å^2^)

**Table:**

	*x*	*y*	*z*	*U*_iso_*/*U*_eq_	
S1	0.18898 (4)	0.33147 (3)	0.40368 (3)	0.01707 (12)	
S2	0.36737 (4)	0.51413 (3)	0.34551 (3)	0.01997 (12)	
O1	1.14563 (12)	0.43927 (8)	0.07886 (8)	0.0191 (2)	
O2	0.73950 (13)	0.50561 (8)	0.24846 (8)	0.0175 (2)	
H2A	0.775 (2)	0.5643 (17)	0.2477 (15)	0.034 (5)*	
O3	0.63540 (13)	0.19012 (8)	0.25602 (8)	0.0211 (2)	
N1	0.55185 (14)	0.35185 (9)	0.27860 (9)	0.0141 (2)	
H1A	0.571 (2)	0.4154 (15)	0.2769 (13)	0.025 (5)*	
N2	0.43294 (14)	0.31214 (9)	0.32068 (9)	0.0154 (3)	
C1	0.78058 (16)	0.32991 (10)	0.20495 (10)	0.0127 (3)	
C2	0.82146 (17)	0.43652 (10)	0.20485 (10)	0.0136 (3)	
C3	0.94371 (17)	0.46929 (11)	0.16183 (10)	0.0147 (3)	
H3A	0.9696	0.5394	0.1619	0.018*	
C4	1.02795 (16)	0.39806 (11)	0.11852 (10)	0.0150 (3)	
C5	0.98999 (17)	0.29255 (11)	0.11710 (10)	0.0157 (3)	
H5A	1.0459	0.2447	0.0877	0.019*	
C6	0.86766 (17)	0.26095 (11)	0.16032 (10)	0.0147 (3)	
H6A	0.8423	0.1907	0.1596	0.018*	
C7	1.23586 (18)	0.36914 (12)	0.03325 (12)	0.0214 (3)	
H7A	1.3118	0.4074	0.0059	0.032*	
H7B	1.1626	0.3318	−0.0190	0.032*	
H7C	1.2938	0.3211	0.0822	0.032*	
C8	0.65227 (16)	0.28531 (11)	0.24849 (10)	0.0139 (3)	
C9	0.34330 (17)	0.37829 (11)	0.35163 (10)	0.0145 (3)	
C10	0.19836 (17)	0.19372 (11)	0.37997 (11)	0.0173 (3)	
H10A	0.1809	0.1812	0.3090	0.021*	
H10B	0.3044	0.1666	0.4134	0.021*	
C11	0.06688 (18)	0.14095 (12)	0.41920 (11)	0.0220 (3)	
H11A	0.0628	0.0687	0.4021	0.033*	
H11B	−0.0363	0.1725	0.3900	0.033*	
H11C	0.0909	0.1483	0.4903	0.033*	
C12	0.19060 (17)	0.56936 (11)	0.37838 (11)	0.0186 (3)	
H12A	0.1653	0.6353	0.3444	0.022*	
H12B	0.0985	0.5237	0.3543	0.022*	
C13	0.2116 (2)	0.58643 (13)	0.48938 (12)	0.0253 (3)	
H13A	0.1168	0.6202	0.5006	0.038*	
H13B	0.3054	0.6291	0.5146	0.038*	
H13C	0.2260	0.5208	0.5231	0.038*	

### Atomic displacement parameters (Å^2^)

**Table:**

	*U*^11^	*U*^22^	*U*^33^	*U*^12^	*U*^13^	*U*^23^
S1	0.0167 (2)	0.01427 (19)	0.0235 (2)	0.00006 (13)	0.01113 (15)	0.00050 (13)
S2	0.0224 (2)	0.01129 (19)	0.0307 (2)	0.00183 (14)	0.01520 (16)	0.00151 (14)
O1	0.0207 (5)	0.0153 (5)	0.0264 (6)	−0.0009 (4)	0.0156 (4)	−0.0009 (4)
O2	0.0195 (5)	0.0094 (5)	0.0276 (6)	−0.0009 (4)	0.0135 (4)	−0.0018 (4)
O3	0.0208 (5)	0.0106 (5)	0.0367 (6)	−0.0004 (4)	0.0161 (5)	0.0010 (4)
N1	0.0136 (6)	0.0098 (6)	0.0214 (6)	−0.0017 (5)	0.0091 (5)	0.0004 (5)
N2	0.0143 (6)	0.0147 (6)	0.0195 (6)	−0.0011 (5)	0.0085 (5)	0.0010 (5)
C1	0.0111 (6)	0.0128 (7)	0.0140 (6)	0.0004 (5)	0.0027 (5)	0.0004 (5)
C2	0.0143 (6)	0.0125 (7)	0.0135 (6)	0.0020 (5)	0.0026 (5)	−0.0002 (5)
C3	0.0171 (7)	0.0112 (6)	0.0160 (7)	−0.0008 (5)	0.0043 (5)	0.0001 (5)
C4	0.0140 (7)	0.0180 (7)	0.0137 (6)	−0.0007 (6)	0.0048 (5)	0.0023 (5)
C5	0.0163 (7)	0.0148 (7)	0.0174 (7)	0.0018 (5)	0.0069 (6)	−0.0019 (5)
C6	0.0167 (7)	0.0102 (6)	0.0173 (7)	0.0000 (5)	0.0044 (5)	−0.0005 (5)
C7	0.0212 (8)	0.0198 (7)	0.0284 (8)	0.0029 (6)	0.0162 (6)	−0.0001 (6)
C8	0.0125 (7)	0.0134 (7)	0.0154 (6)	−0.0001 (5)	0.0024 (5)	−0.0001 (5)
C9	0.0148 (7)	0.0124 (7)	0.0164 (6)	−0.0002 (5)	0.0043 (5)	0.0018 (5)
C10	0.0175 (7)	0.0136 (7)	0.0221 (7)	−0.0015 (6)	0.0073 (6)	−0.0003 (6)
C11	0.0207 (8)	0.0219 (8)	0.0242 (8)	−0.0059 (6)	0.0071 (6)	0.0020 (6)
C12	0.0170 (7)	0.0144 (7)	0.0242 (7)	0.0042 (6)	0.0048 (6)	−0.0018 (6)
C13	0.0302 (9)	0.0225 (8)	0.0259 (8)	−0.0003 (7)	0.0121 (7)	−0.0043 (6)

### Geometric parameters (Å, º)

**Table:**

S1—C9	1.7485 (14)	C4—C5	1.397 (2)
S1—C10	1.8115 (15)	C5—C6	1.380 (2)
S2—C9	1.7678 (15)	C5—H5A	0.9300
S2—C12	1.8151 (15)	C6—H6A	0.9300
O1—C4	1.3591 (17)	C7—H7A	0.9600
O1—C7	1.4276 (17)	C7—H7B	0.9600
O2—C2	1.3588 (17)	C7—H7C	0.9600
O2—H2A	0.82 (2)	C10—C11	1.516 (2)
O3—C8	1.2430 (17)	C10—H10A	0.9700
N1—C8	1.3448 (18)	C10—H10B	0.9700
N1—N2	1.3804 (16)	C11—H11A	0.9600
N1—H1A	0.836 (19)	C11—H11B	0.9600
N2—C9	1.2837 (18)	C11—H11C	0.9600
C1—C6	1.3932 (19)	C12—C13	1.519 (2)
C1—C2	1.4176 (19)	C12—H12A	0.9700
C1—C8	1.4827 (19)	C12—H12B	0.9700
C2—C3	1.382 (2)	C13—H13A	0.9600
C3—C4	1.387 (2)	C13—H13B	0.9600
C3—H3A	0.9300	C13—H13C	0.9600
			
C9—S1—C10	101.15 (7)	H7B—C7—H7C	109.5
C9—S2—C12	105.35 (7)	O3—C8—N1	120.63 (13)
C4—O1—C7	117.17 (11)	O3—C8—C1	121.87 (12)
C2—O2—H2A	111.7 (14)	N1—C8—C1	117.49 (12)
C8—N1—N2	118.51 (12)	N2—C9—S1	118.17 (11)
C8—N1—H1A	118.4 (13)	N2—C9—S2	123.89 (11)
N2—N1—H1A	122.7 (13)	S1—C9—S2	117.94 (8)
C9—N2—N1	116.59 (12)	C11—C10—S1	107.85 (10)
C6—C1—C2	117.53 (12)	C11—C10—H10A	110.1
C6—C1—C8	116.99 (12)	S1—C10—H10A	110.1
C2—C1—C8	125.48 (12)	C11—C10—H10B	110.1
O2—C2—C3	120.66 (12)	S1—C10—H10B	110.1
O2—C2—C1	118.91 (12)	H10A—C10—H10B	108.4
C3—C2—C1	120.42 (13)	C10—C11—H11A	109.5
C2—C3—C4	120.25 (13)	C10—C11—H11B	109.5
C2—C3—H3A	119.9	H11A—C11—H11B	109.5
C4—C3—H3A	119.9	C10—C11—H11C	109.5
O1—C4—C3	114.98 (12)	H11A—C11—H11C	109.5
O1—C4—C5	124.37 (13)	H11B—C11—H11C	109.5
C3—C4—C5	120.65 (13)	C13—C12—S2	114.24 (11)
C6—C5—C4	118.46 (13)	C13—C12—H12A	108.7
C6—C5—H5A	120.8	S2—C12—H12A	108.7
C4—C5—H5A	120.8	C13—C12—H12B	108.7
C5—C6—C1	122.69 (13)	S2—C12—H12B	108.7
C5—C6—H6A	118.7	H12A—C12—H12B	107.6
C1—C6—H6A	118.7	C12—C13—H13A	109.5
O1—C7—H7A	109.5	C12—C13—H13B	109.5
O1—C7—H7B	109.5	H13A—C13—H13B	109.5
H7A—C7—H7B	109.5	C12—C13—H13C	109.5
O1—C7—H7C	109.5	H13A—C13—H13C	109.5
H7A—C7—H7C	109.5	H13B—C13—H13C	109.5
			
C8—N1—N2—C9	177.63 (12)	C8—C1—C6—C5	179.98 (12)
C6—C1—C2—O2	−179.53 (12)	N2—N1—C8—O3	1.9 (2)
C8—C1—C2—O2	0.6 (2)	N2—N1—C8—C1	−179.09 (11)
C6—C1—C2—C3	−0.1 (2)	C6—C1—C8—O3	8.5 (2)
C8—C1—C2—C3	−179.96 (12)	C2—C1—C8—O3	−171.64 (13)
O2—C2—C3—C4	179.25 (12)	C6—C1—C8—N1	−170.50 (12)
C1—C2—C3—C4	−0.2 (2)	C2—C1—C8—N1	9.4 (2)
C7—O1—C4—C3	−179.94 (12)	N1—N2—C9—S1	179.01 (9)
C7—O1—C4—C5	−0.2 (2)	N1—N2—C9—S2	−1.42 (18)
C2—C3—C4—O1	−179.83 (12)	C10—S1—C9—N2	−6.72 (13)
C2—C3—C4—C5	0.4 (2)	C10—S1—C9—S2	173.68 (8)
O1—C4—C5—C6	179.87 (12)	C12—S2—C9—N2	170.45 (12)
C3—C4—C5—C6	−0.4 (2)	C12—S2—C9—S1	−9.98 (10)
C4—C5—C6—C1	0.1 (2)	C9—S1—C10—C11	−178.68 (10)
C2—C1—C6—C5	0.1 (2)	C9—S2—C12—C13	88.23 (12)

### Hydrogen-bond geometry (Å, º)

**Table:**

*D*—H···*A*	*D*—H	H···*A*	*D*···*A*	*D*—H···*A*
O2—H2*A*···O3^i^	0.82 (2)	1.80 (2)	2.6118 (15)	177 (2)
N1—H1*A*···O2	0.836 (19)	1.957 (19)	2.6384 (16)	137.9 (17)
C3—H3*A*···O3^i^	0.93	2.52	3.1975 (18)	130

Symmetry code: (i) −*x*+3/2, *y*+1/2, −*z*+1/2.
