# Supplementary figures and images for: Crystal structure of N′-[bis­(ethyl­sulfan­yl)methyl­idene]-2-hy­droxy-4-meth­oxy­benzohydrazide
Source: Acta Crystallogr E Crystallogr Commun. 2015 Nov 21;71(Pt 12):o967–8. doi: 10.1107/S2056989015021271 (PMC4719926; doi:10.1107/S2056989015021271)

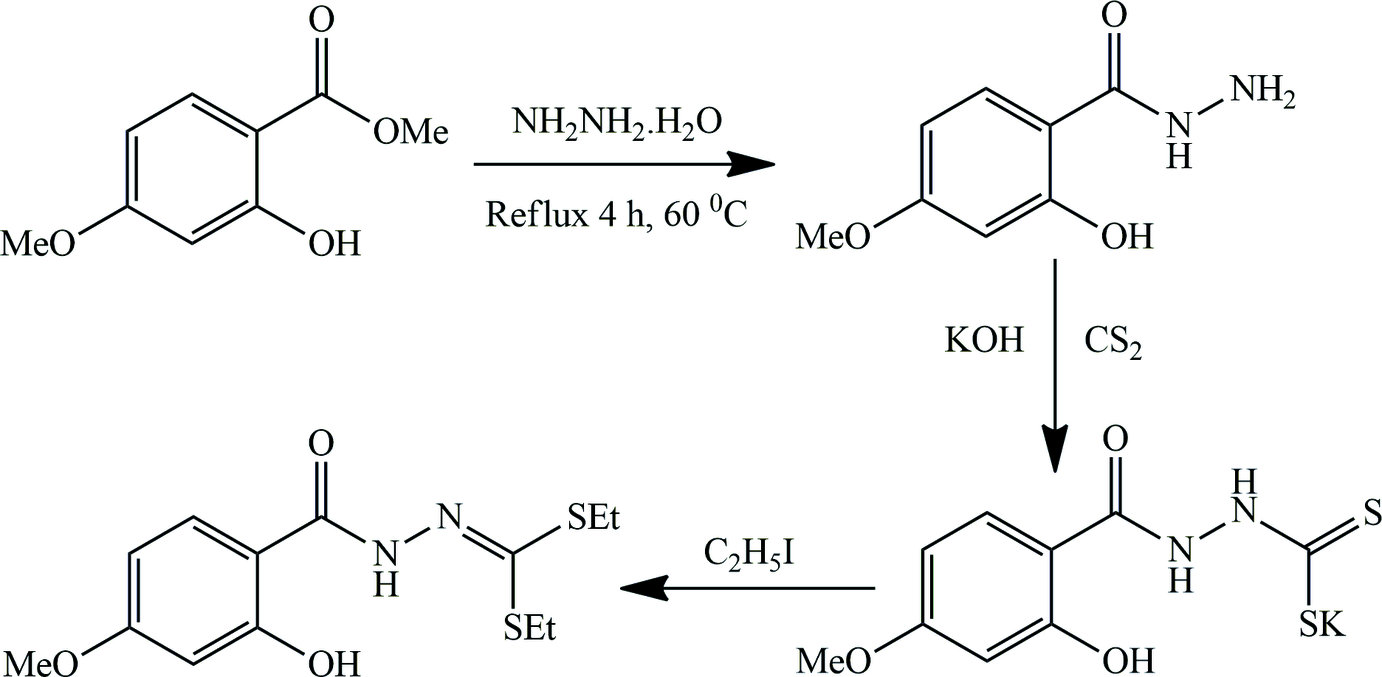

Supplement: Supplementary file 4 [file e-71-0o967-fig1.tif]

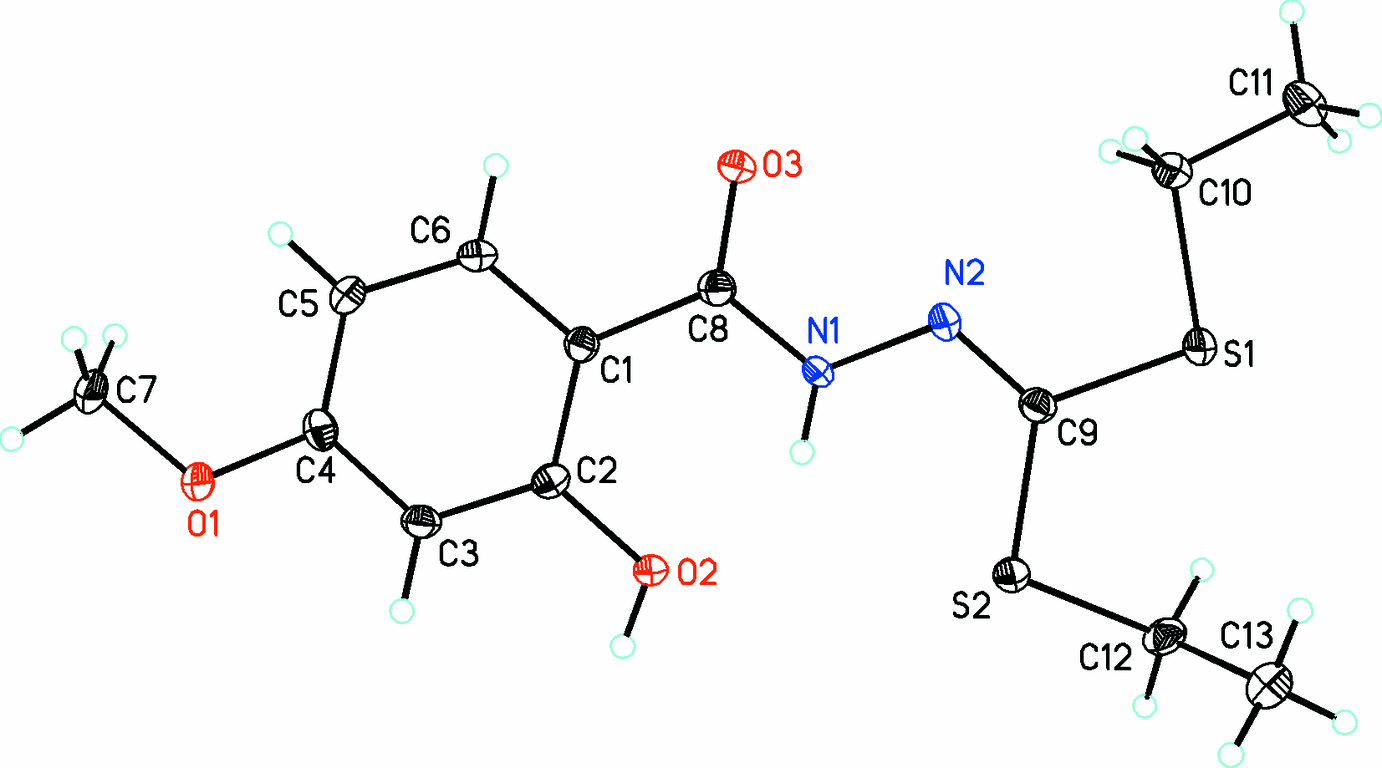

Supplement: Supplementary file 5 [file e-71-0o967-fig2.tif]

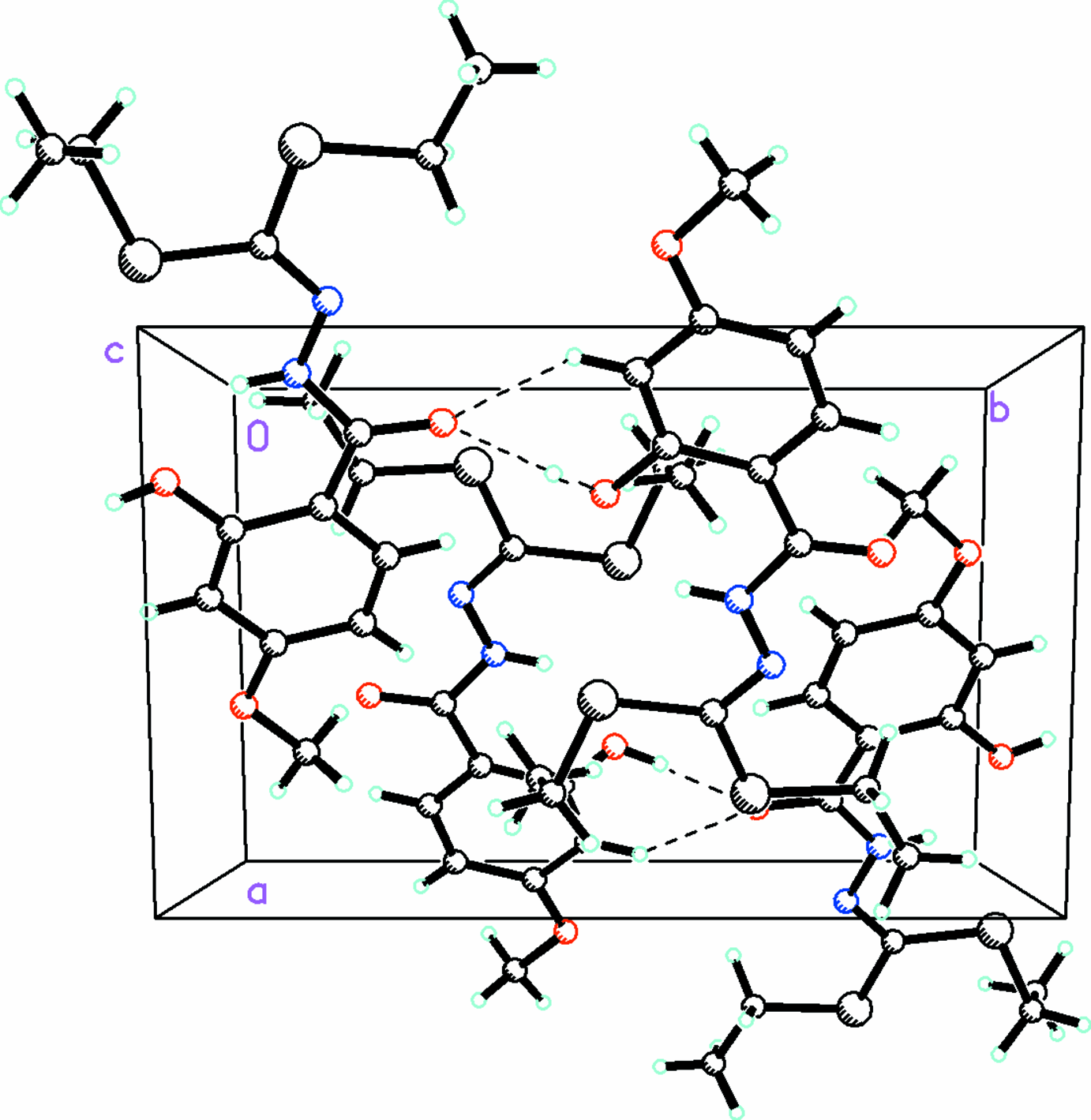

Supplement: Supplementary file 6 [file e-71-0o967-fig3.tif]
